# Combined associations of hs-CRP and cognitive function with all-cause mortality among oldest-old adults in Chinese longevity areas: a prospective cohort study

**DOI:** 10.1186/s12979-019-0170-y

**Published:** 2019-11-17

**Authors:** Chen Chen, Yingchun Liu, Zhaojin Cao, Zhaoxue Yin, Feng Zhao, Yuebin Lv, Zuyun Liu, Chen Mao, Shixun Song, Ling Liu, Yingli Qu, Saisai Ji, Jun Duan, Jiaonan Wang, Virginia Byers Kraus, Yi Zeng, Xiaoming Shi

**Affiliations:** 1 National Institute of Environmental and Health, Chinese Center for Disease Control and Prevention, Bejing, 100021 People’s Republic of China; 2 Division of Non-communicable Disease and Healthy Ageing Management, Chinese Center for Disease Control and Prevention, Beijing, 102206 China; 3 Department of Pathology, Yale School of Medicine, New Haven, CT 06511 USA; 4 Division of Epidemiology, School of Public Health, Southern Medical University, Guangzhou, 510515 Guangdong China; 5 Center for Global Health, School of Public Health, Nanjing Medical University, Nanjing, 211166 Jiangsu China; 6 Duke Molecular Physiology Institute and Department of Medicine, Duke University School of Medicine, Durham, North Carolina, 27711 USA; 7 Center for the study of Aging and Human Development and the Geriatric Division, School of Medicine, Duke University, Durham, North Carolina, 27711 USA; 8 Center for Healthy Aging and Development Studies, National School of Development, Peking University, Beijing, 100871 China

**Keywords:** Hs-CRP, Cognition, Mortality, Oldest-old

## Abstract

**Background:**

Inflammatory markers, such as high sensitivity C-reactive protein (hs-CRP), and cognitive impairment (CI) are associated with mortality; CRP is related to the deterioration of CI. However, it is still unknown whether these two indices predict mortality independent of each other. Furthermore, their joint effect on all-cause mortality has not been well established, especially in oldest-old adults.

**Methods:**

Based on data from the 2012 wave of the Chinese Longitudinal Healthy Longevity Survey (CLHLS), we included 1447 oldest-old adults (mean age 84.7 years and 58.7% were female, weighted) with information on hs-CRP (stratified by a cutoff value of 3.0 mg/L) and cognition (quantified by Mini-Mental Status Examination (MMSE) scored according to the personal educational level) at baseline. Mortality was assessed in followed 2014 and 2017 waves. Cox proportional hazards regression models were used, with adjustment for hs-CRP and cognition (mutually controlled) and several traditional mortality risk factors.

**Results:**

During a median follow-up period of 32.8 months (Q1-Q3, 9.7–59.0 months), 826 participants died. Hs-CRP [HR _> 3.0 mg/L vs ≤ 3.0 mg/L_: 1.64 (95% CI, 1.17, 2.30)] and cognition [HR _CI vs normal_: 2.30 (95% CI, 1.64, 3.21)] each was independent predictor of all-cause mortality, even after accounting for each other and other covariates. Monotonic and positive associations were observed in combined analyses, in which the highest mortality risk was obtained in elders with both high hs-CRP_> 3.0 mg/L_ and CI [HR: 3.56 (95% CI, 2.35, 5.38)].The combined effects were stronger in male and younger oldest-old (aged 80–89 years).

**Conclusion:**

High hs-CRP and CI, both individually and jointly, were associated with increased all-cause mortality risks in Chinese oldest-old. Intervention strategies for preventing inflammation and maintaining adequate cognitive function may be more important in male and younger oldest-old for reducing mortality risk.

## Background

C-reactive protein (CRP) is an acute-phase reactant that is a strong marker for underlying systemic inflammation. Based on laboratory and epidemiologic data, inflammation promotes both the initiation and the progression of atherosclerosis [1, 2]. Elevated levels of CRP are associated with increased risk of cardiovascular events [3–5] and mortality [6, 7]. This association is mostly apparent with CRP levels of > 3.0 mg/L [8], which has become a well-established classification criterion for predicting high risk level of cardiovascular disease (CVD) [9, 10]. However, the high risk threshold value varies across different age groups. Though higher levels of CRP represent a risk factor for all-cause mortality in both middle-aged [11, 12], 65 -[13] and 75-year-old [14] cohorts, this association is attenuated in 80- and 85-year-old cohorts [15]. Furthermore, data about the epidemiology and predictive value of CRP, especially in oldest-old (aged ≥80 years), are sparse [15, 16]; the two previous studies of this age group were limited to less than 300 older persons each. Therefore, the predictive value of elevated CRP on mortality risk needs further evaluation in oldest-old adults aged 80 years or older.

Cognitive decline is often associated with ageing, and even mild levels of cognitive impairment (CI) has been associated with increased risk of mortality in the elderly [17]. Inflammatory markers, such as CRP, have been found in the β-amyloid plaques and neurofibrillary tangles in patients with dementia or CI [18, 19]. It is still unclear whether participants with high CRP level are also those with poor cognitive function [20, 21]. Moreover, to our knowledge, little is known about the combined effects of CRP level and cognitive function on all-cause mortality risk, especially in the oldest-old adults. It is also unknown whether these two indices predict mortality independent of each other.

In this study, we hypothesized that high sensitivity CRP (hs-CRP) level and cognitive function, both individually and jointly, are associated with the length of remaining life in Chinese elderly. To evaluate this hypothesis we investigated the relationship between hs-CRP and cognitive performance with all-cause mortality in the oldest-old using a large population-based cohort of participants aged 80 years or older who were followed up for 5 years.

## Methods

### Study population

Participants for this study were ascertained from the 8 longevity areas during the sixth wave of the Chinese Longitudinal Healthy Longevity Survey (CLHLS) in 2012. The 8 areas represented 1/3 of the longevity areas selected by the Chinese Society of Gerontology in 2011 [22]. Compared with other areas, longevity areas have higher densities of oldest old adults, especially for centenarians (> 7/100, 000), and higher life expectancies. A total of 1535 participants aged 80 years or older were enrolled in the baseline survey, including 555 octogenarians, 461 nonagenarians and 519 centenarians. Details of the study design and its sampling method have been described previously [23]. After exclusion of 29 subjects due to missing data on hs-CRP value and 59 subjects on cognition, a total of 1447 oldest-old adults were included in the final analysis. The followed two waves of the survey were carried out in 2014 and 2017. The study was approved by the Ethics Committee of Peking University and Duke University. All participants signed written informed consent.

### High sensitivity CRP

Overnight fasting blood samples were collected from all participants. Plasma levels of hs-CRP were measured by immunoturbidimetric assay (Roche Diagnostic, Mannheim, Germany) using an Automatic Biochemistry Analyzer (Hitachi 7180, Japan). All laboratory analyses were conducted by the central clinical lab at Capital Medical University in Beijing. The minimal detectable concentration of hs-CRP was 0.11 mg/L. Quality control measures in the laboratory were described previously [24]. We used both hs-CRP quartiles and a binary variable (the cutoff value was 3.0 mg/L) for the individual association analyses and applied the binary variable in the combined association analyses.

### Cognition

The Mini-Mental Status Examination (MMSE), which has been a widely used 30-point assessment tool for screening cognitive impairment, was administrated to all participants. The designation of cognitive impairment was based on the MMSE score taking into account the educational level [25, 26]: participants with an MMSE score less than 18 and no formal schooling, or those with an MMSE score less than 24 and with at least 1 year of formal schooling were defined as having cognitive impairment; otherwise, those who failed to meet these criteria were defined as having normal cognition.

### Date of death

The date of death was collected from official death certificates when available; otherwise, the next-of-kin and local village doctor were consulted. Survival time (in months) was calculated from the interview date at baseline until the date of death (for participants who died), the interview date of follow-up survey (for participants who were alive), or administrative censoring date (i.e., the middle date between the last survey when the participant was interviewed and the subsequent survey, for participants who were lost to follow-up), whichever came first.

### Covariates

Sociodemographic characteristics included age, sex (male vs female), education (any formal education, defined as at least 1 year of formal schooling), and current marital status (currently married vs other). Data collected on health behaviors and characteristics included current smoking, current alcohol consuming, regular exercise (yes vs no), and being able to get adequate medical service for any illness (yes vs no). Self-reported history of chronic diseases included hypertension, diabetes mellitus, heart disease, stroke and cerebrovascular disease, respiratory disease and cancer. Physical examination data included weight and height (for body mass index (BMI) calculated as weight in kilograms divided by height in meters squared), and waist circumference (WC) collected using standardized measurement protocols [27]. Central obesity (yes vs no) was defined as WC ≥ 85 cm in men and WC ≥ 80 cm in women. These covariates have been well defined and studied in CLHLS, showing important effects both on hs-CRP level [24] and mortality [28].

### Statistical analyses

In all analyses, we adopted a survey weight variable that was constructed according to the distribution of age, sex, and urban/rural residence in the Chinese population in the survey year [23]. To observe the combined effects of the inflammation marker and cognition on mortality, we categorized hs-CRP and cognition into binary variables and created a 4-level joint hs-CRP/cognition variable that included the following groups:

- Group 1: hs-CRP ≤ 3.0 mg/L and normal cognition
- Group 2: hs-CRP > 3.0 mg/L and normal cognition
- Group 3: hs-CRP ≤ 3.0 mg/L and cognitive impairment
- Group 4: hs-CRP > 3.0 mg/L and cognitive impairment

Characteristics of the study population were presented as means (± standard deviation [SD]) or percentages in the full sample and by the 4 combined groups. Kaplan-Meier analysis was used to compare survival curves, and log-rank test was used to assess significance. The individual associations of hs-CRP levels with mortality without and with adjustment for cognitive impairment was analyzed (and vice versa). The combined associations of hs-CRP/cognition groups with risk of mortality were then performed according to the above 4 groups. We added an interaction term, inflammation marker*cognition, in models with hs-CRP and cognitive impairment included as independent to examine whether the association of hs-CRP levels with risk of death was modified by cognition groups (and vice versa).

Two weighted Cox proportional hazards models were used with the outcome of all-cause mortality. Model 1 was adjusted for age and sex; model 2 additionally adjusted for education, drinking, smoking, marital status, regular exercise, medication, BMI, central obesity, self-reported history of hypertension, diabetes mellitus, heart disease, stroke and cerebrovascular disease, respiratory disease and cancer; Education was not included in model 2 when cognition and the combined hs-CRP/cognition variable were analyzed because we defined cognition status according to personal education levels. We documented the Hazard ratios (HRs) and 95% confidence intervals (CIs). We also assessed the ability of specific variables to predict mortality by estimating the Harrell’s C-statistics [29].

Next, we repeated the above analysis for two subgroups: age (80–89 years vs. ≥90 years) and sex (female vs. male). The interactions between age, sex and the 4-level joint variable for mortality risk were evaluated in model 2 to determine whether the joint effect was the same in each subgroup.

To test the robustness of our results, we performed several sensitivity analyses. First, we repeated models with replacement of missing data on sociodemographic factors using mean imputation techniques. Second, we repeated models by excluding the participants with extreme high hs-CRP levels (> 10 mg/L), generally considered an indication of infection [30]. Third, we excluded individuals who died in the first two years to avoid the confounding of mortality due to preexisting disease. Fourth, we reran the combined models without adjustment for several chronic diseases in order to avoid unfavorable over-adjusted estimates. All statistical analyses were performed using SAS version 9.4 (SAS Institute, Cary, NC). *P* < 0.05 (two-tailed) was considered statistically significant.

## Results

### Basic characteristics of study participants

**Fig. 1:**
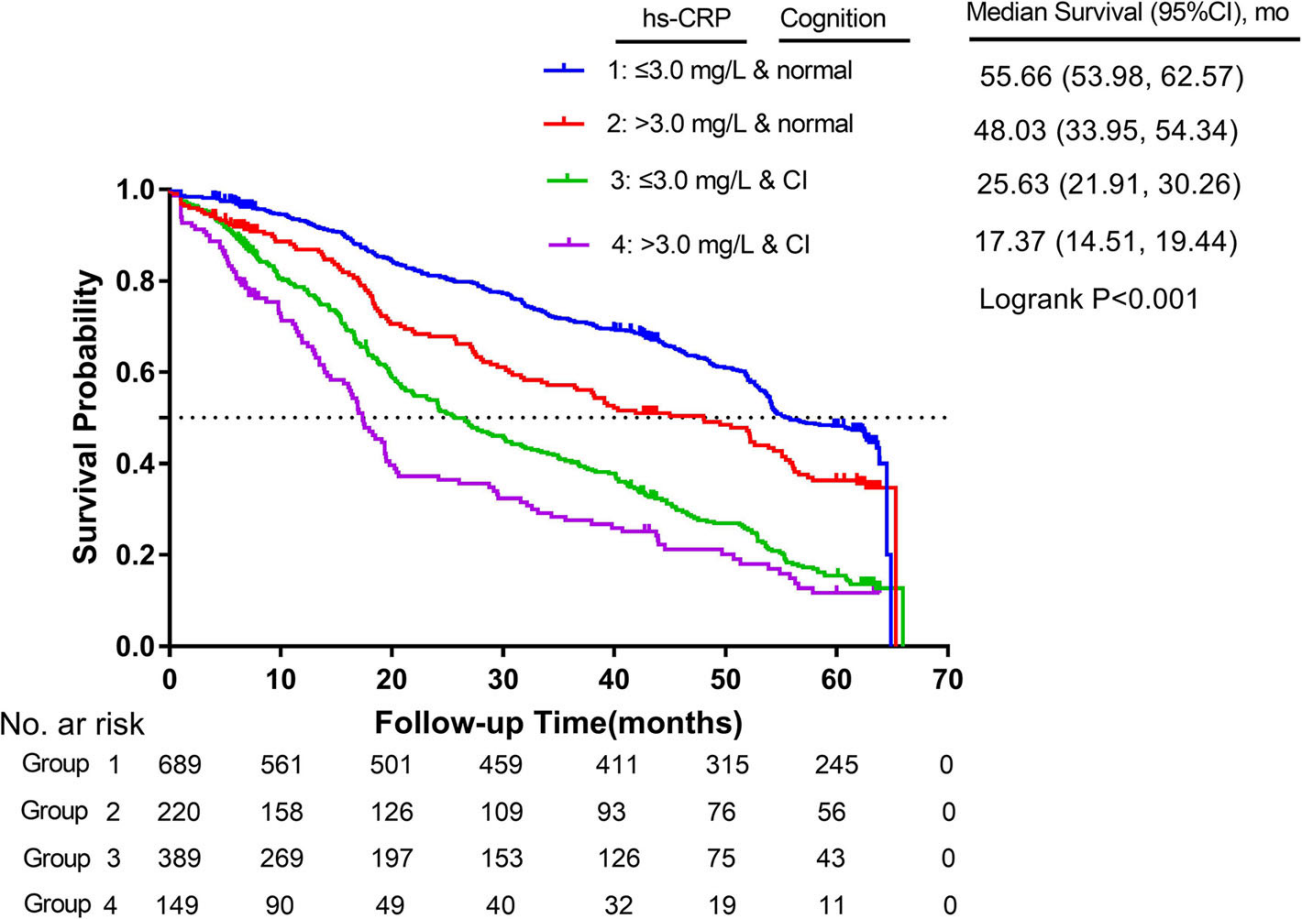
Kaplan-Meier survival curves for Chinese oldest-old stratified by the 4-level joint hs-CRP/cognition groups (*N* = 1447). Group 1 (hs-CRP ≤ 3.0 mg/L and normal cognition), group 2 (hs-CRP > 3.0 mg/L and normal cognition), group 3 (hs-CRP ≤ 3.0 mg/L and CI), group 4 (hs-CRP > 3.0 mg/L and CI)

During an unweighted median follow-up period of 32.8 months (Q1-Q3, 9.7–59.0 months); a total of 826 participants died, 287 were lost to follow-up, and 334 survived. The median level of hs-CRP was 1.07 mg/L; 9.1% (*n* = 137) of the participants had hs-CRP levels higher than 10 mg/L. The median MMSE score was 23.0; the prevalence of CI was 37.2% (28.2% for males and 42.1% for females). We did not find significant differences in the baseline characteristics between individuals who were lost to follow-up and those who were remained in the study, except that a higher proportion of regular exercise was found in the former group (Additional file 1: Table S2). Table 1 lists the characteristics of all study participants across the 4 mutually exclusive hs-CRP/cognition groups. The mean (SD) age of the 1447 participants at baseline was 85 (0.2) years (weighted, hereafter); and 59% (*n* = 899) were female. Participants with both higher hs-CRP (> 3.0 mg/L) and worse cognition were older, were less likely to take regular exercise, smoke, drink, and currently married (P for trend all< 0.05, Table 1), and more likely to suffer with diabetes, stroke and cerebrovascular disease (P for trend all< 0.05, Additional file 1: Table S1). Kaplan-Meier survival curves for these 4 groups separated early and the separation persisted throughout the subsequent 5 years (log-rank test for trend < 0.01, Fig. 1). The median survival time from group 1 to 4 were 55.7, 48.0, 25.6 and 17.4 months, respectively.

**Table 1:** Baseline characteristics of study participants across the 4 groups (*N* = 1447)

Characteristic	Overall^a^ (*n* = 1447)	hs-CRP ≤ 3.0 mg/L & normal cognition (*n* = 689)	hs-CRP > 3.0 mg/L & normal cognition (*n* = 220)	hs-CRP ≤ 3.0 mg/L & CI (*n* = 389)	hs-CRP > 3.0 mg/L & CI (*n* = 149)	P for trend
Age, years, mean (SD)	84.7 (0.2)	84.3 (0.2)	83.9 (0.3)	86.9 (0.7)	86.5 (0.8)	< 0.001
Female	899 (58.7)	417 (58.2)	115 (52.5)	277 (72.2)	90 (54.7)	0.369
≥1 Years of education	326 (30.8)	175 (31.4)	54 (35.2)	71 (22.1)	26 (29.6)	0.385
Currently married, yes^b^	297 (37.9)	195 (41.5)	55 (44.5)	31 (15.5)	16 (23.3)	< 0.001
Regular exercise, yes^b^	182 (16.1)	119 (19.3)	27 (14.5)	28 (8.4)	8 (1.6)	< 0.001
Current smoking, yes^b^	151 (11.8)	83 (14.0)	28 (9.1)	28 (6.9)	12 (6.6)	0.014
Current alcohol drinking, yes^b^	169 (13.0)	97 (15.5)	23 (9.5)	35 (8.3)	14 (6.4)	0.014
Body mass index, kg/m^2^, mean (SD)	21.4 (0.3)	21.3 (0.3)	20.7 (0.5)	23.2 (2.0)	21.4 (0.8)	0.424
Central obesity, yes^bc^	465 (39.8)	243 (40.0)	74 (41.6)	120 (38.4)	28 (34.6)	0.716
Adequate medical service, yes^b^	1311 (94.2)	643 (94.0)	201 (96.0)	342 (91.9)	125 (94.7)	0.855

Values are given as No. (%) unless otherwise stated. No. was based on study samples (unweighted). Means (SD) and percentages were weighted population estimates. CI, cognitive impairment

^a^Of 1447 participants, 46 with missing data on the weight variable were excluded for calculating weighted population estimates

^b^Numbers of missing data ranged from 3 to 41 (8 for married status, 41 for regular exercise, 8 for current smoking, 3 for alcohol drinking, 18 for central obesity, and 6 for adequate medication)

^c^Central obesity (yes vs no) was defined as waist circumference (WC) ≥ 85 cm in men and WC ≥ 80 cm in women

### Individual associations of hs-CRP and cognitive status with all-cause mortality

Table 2 shows the associations of hs-CRP levels with all-cause mortality. After adjustment for age and sex (Model 1), there was a graded association between higher hs-CRP levels and increased mortality that was slightly decreased after consideration of cognition (P for trend all< 0.05). Compared with participants in the lowest hs-CRP quartile group, those in the highest quartile group had a nearly 2-fold increased risk for death [HR Q4 vs Q1: 2.02 (95% CI, 1.25, 3.26)]. After additional adjustment for smoking, marital status, regular exercise, central obesity, self-reported history of hypertension, respiratory diseases, and cancer (Model 2), and after accounting for differences in cognitive status, the association between hs-CRP levels with mortality was slightly more pronounced [HR Q4 vs Q1: 2.10 (95% CI, 1.30, 3.39)] (P-trend< 0.05). Similar statistically significant results were obtained when hs-CRP was categorized by the cutoff value of 3.0 mg/L (Table 2) and when assessed as a continuous variable (Additional file 1: Table S2).

**Table 2:** Hazard ratios for the individual associations of hs-CRP levels and cognitive status with all-cause mortality (*N* = 1447)

hs-CRP (mg/L)	Model 1	Model 1 + cognition	Model 2	Model 2 + cognition^a^
Cut-off at 3.0 mg/L				
Low hs-CRP (≤3.0 mg/L)	1	1	1	1
High hs-CRP (> 3.0 mg/L)	1.75 (1.30, 2.37)	1.67 (1.19, 2.34)	1.76 (1.30, 2.37)	1.64 (1.17, 2.30)
Cut-offs by quartiles				
Q1(< 0.41)	1	1	1	1
Q2(0.41–1.05)	1.32 (0.87, 2.01)	1.36 (0.87, 2.12)	1.49 (0.95, 2.35)	1.47 (0.94, 2.30)
Q3(1.06–3.05)	1.24 (0.82, 1.89)	1.14 (0.71, 1.83)	1.40 (0.89, 2.19)	1.19 (0.73, 1.92)
Q4(≥3.06)	2.20 (1.45, 3.32)	2.02 (1.25, 3.26)	2.39 (1.53, 3.73)	2.10 (1.30, 3.39)
P for trend	< 0.001	0.009	< 0.001	0.008
Cognition groups	Model 1	Model 1 + hs-CRP	Model 2^a^	Model 2 + hs-CRP^a^
Normal cognition	1	1	1	1
Cognitive impairment	2.72 (1.90, 3.91)	2.29 (1.63, 3.21)	2.73 (1.91, 3.91)	2.30 (1.64, 3.21)

Model 1 adjusted for age and sex; model 2 further adjusted for education, drinking, smoking, marital status, regular exercise, medication, BMI, central obesity, self-reported history of hypertension, diabetes mellitus, heart disease, stroke and cerebrovascular disease, respiratory disease and cancer

^a^Education was not included

Similarly, after adjustment for multiple mortality risk factors and consideration of differences in baseline hs-CRP levels, poorer cognitive function (CI) was associated with higher risk of mortality [HR CI vs normal: 2.30 (95% CI, 1.64, 3.21)] (Table 2).

### Combined associations of hs-CRP and cognitive status on all-cause mortality

**Fig. 2:**
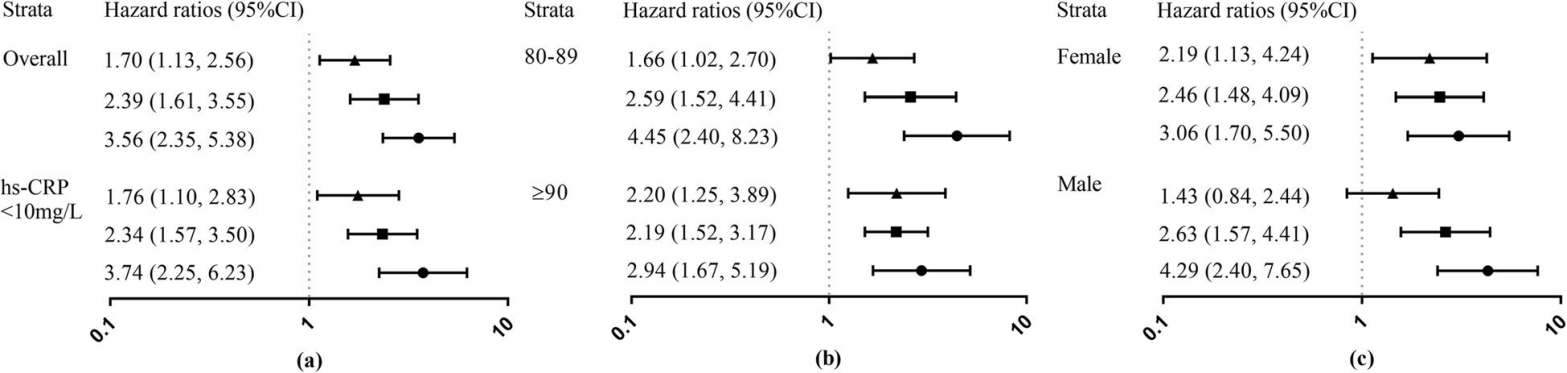
HRs for the combined associations of hs-CRP and CI with all-cause mortality according to sensitivity analysis (Panel **a**) and specified subgroups, stratified by age (< vs. ≥ 90 y; Panel **b**) and sex (Panel **c**). All models were adjusted for age (not in Panel **b**) and sex (not in Panel **c**), drinking, smoking, marital status, regular exercise, medication, BMI, central obesity, self-reported history of hypertension, diabetes mellitus, heart disease, stroke and cerebrovascular disease, respiratory disease and cancer. Group 1 (hs-CRP ≤ 3.0 mg/L and normal cognition), group 2 (hs-CRP > 3.0 mg/L and normal cognition), group 3 (hs-CRP ≤ 3.0 mg/L and CI), group 4 (hs-CRP > 3.0 mg/L and CI). Group symbols are as follows: ▲ group 2, ■ group 3, ● group 4), the estimated HRs for each group are compared with group 1 (HR = 1.0, not shown)

Monotonic and positive associations were observed in combined analyses, the higher the joint hs-CRP/cognition group from group1 to group 4, the higher the risk of death (P-trend all< 0.01, Table 3; Fig. 2a). In addition, the joint analysis suggested the presence of an interaction between hs-CRP and cognitive status for prediction of 5-year mortality (P for interaction< 0.01, Table 3). For instance, among participants with normal cognition, high hs-CRP group (> 3.0 mg/L) was associated with a 1.7-fold [HR Group2 vs. Group1: 1.70 vs. 1.00] increase in the risk for mortality compared to those in the low hs-CRP group (≤3.0 mg/L). However, among participants with CI, the risk estimate was increased only by 49% among elders with high hs-CRP (> 3.0 mg/L) [HR Group4 vs. Group3: 3.56 vs. 2.39]. The Harrell’s C-statistic, for the hs-CRP/cognition combined variable was 0.63 (95%CI, 0.60–0.66), followed by cognitive status 0.61 (95%CI, 0.58–0.64) and hs-CRP 0.54 (95%CI, 0.51–0.58, for quartiles).

**Table 3:** Hazard ratios for the combined associations of hs-CRP and cognitive impairment with all-cause mortality (*N* = 1447)

Groups/HR	No. of deaths	Model 1	Model 2^a^
1: hs-CRP ≤ 3.0 mg/L and normal cognition	312	1	1
2: hs-CRP > 3.0 mg/L and normal cognition	117	1.80 (1.24, 2.61)	1.70 (1.13, 2.56)
3: hs-CRP ≤ 3.0 mg/L and CI	286	2.79 (1.82, 4.28)	2.39 (1.61, 3.55)
4: hs-CRP > 3.0 mg/L and CI	111	4.61 (3.16, 6.72)	3.56 (2.35, 5.38)
P for trend	–	< 0.001	< 0.001

Model 1 adjusted for age and sex; model 2 further adjusted for drinking, smoking, marital status, regular exercise, medication, BMI, central obesity, self-reported history of hypertension, diabetes mellitus, heart disease, stroke and cerebrovascular disease, respiratory disease and cancer. CI = cognitive impairment

^a^ P for interaction< 0.01

### Subgroup and sensitivity analyses

Stratified results generally did not change appreciably in different age and sex subgroups (Fig. 2b and c) except for some of the non-significant effect estimates in the male subgroup. Interestingly, we observed that the estimated mortality risk was stronger in males and octogenarians compared with females, nonagenarians and centenarians (all P for interaction < 0.01).

In sensitivity analyses, we found that: (1) the results were materially unchanged after missing data were imputed; (2) exclusion of individuals who had abnormal hs-CRP values at baseline (*n* = 137) did not affect the associations of hs-CRP, cognitive status and mortality [HR Group4 vs. Group1: 3.74 (2.25–6.23), Fig. 2a]; (3) exclusion of early mortality (individuals who died within the first two years of the study) did not attenuate the risk-mortality relation appreciably; and (4) with and without adjustment for chronic diseases obtained comparable risk estimates on all-cause mortality (Additional file 1: Table S4).

## Discussion

In our study of 1447 Chinese oldest-old adults we found that hs-CRP and cognition were each independent predictors of all-cause mortality, even after accounting for each other and several traditional mortality risk factors. Significant interaction effects were observed in combined analyses and the combined effects were stronger in male and younger oldest-old (aged 80–89 years) for all-cause mortality prediction.

The results of the individual associations of hs-CRP and cognitive status with all-cause mortality were consistent with previous research [8, 17], which showed that both high levels of CRP and poor cognitive function (such as CI) in the oldest-old represent risk factors for mortality, though most of the prior studies have not adequately accounted for each other. For example, in a cohort study [16] of oldest-old aged 90 or older with outcomes being total mortality and incidence of dementia, high levels of CRP (the cutoff value was 5.0 mg/L) were associated with greater risk of total mortality [HR: 1.7 (95% CI, 1.0, 2.9)] after adjustment for APOE4 genotype and other risk factors; however, this study did not consider the impact of cognition on the relationship between CRP exposure and mortality. Another cohort study [31] of 340 centenarians (mean age 101 years) with explanatory variables that included cognition and CRP levels found that higher MMSE scores were related with lower 1-year risk of mortality [HR: 0.978 (95% CI, 0.946, 0.993)] (P each point< 0.01), but they did not consider the influence of CRP levels on the relationship between cognition and mortality. Other long-term follow-up (more than 7 years) prospective studies [32–34] in community-based aged populations also observed that CI was associated with increased risk of mortality; similarly, none of them considered the effects of CRP levels on the association.

To the best of our knowledge, this is the first cohort study to examine the combined effect of CRP and cognition on mortality among oldest-old aged 80 years or older in China. One prior study [13] among 6817 German unselected participants aged 65 years or older found significant interaction effects between age [HR CRP > 3.0 mg/L & ≥75 years: 3.18 (95% CI, 2.69, 3.75)], sex [HR CRP > 3.0 mg/L & male: 2.25 (95% CI, 1.92, 2.63)], and arterial hypertension [HR CRP > 3.0 mg/L & SBP ≥ 140 mmHg: 1.30 (1.10, 1.54)] with CRP (the cutoff value was 3.0 mg/L) on all-cause mortality. Our study extends the previous work by including a more comprehensive consideration of additional mortality risk factors such as a more accurate assessment of inflammation pathway biomarkers and study of a population of oldest-old of selected without consideration of function.

There are several explanations for the potential additive or combined effect of CRP levels and cognitive limitations on mortality risk in older adults. Firstly, oldest-old individuals with cognitive decline may be more likely to have financial difficulties and therefore less able to manage their health [35, 36] and access treatment (the proportion with a better economic status was lower in the CI group in our study, data not shown) when they have a preclinical symptom such as inflammation, thereby increasing their risk of long-term comorbidity [37] and related mortality. Secondly, elevated CRP levels were associated with greater cognitive decline [20, 38], which indicates that inflammatory mechanisms may contribute to CI, though it is unclear whether peripheral inflammation is a by-product of neuropathology or whether it directly contributes to cognitive damage [39]. In this case, persons with combined risk indicators of both high hs-CRP and CI, might be expected to have a higher degree of inflammation and thereby a greater risk of mortality than those with elevation of a single components.

In our study, the 4-level joint hs-CRP/cognition variable appeared to be more sensitive in male and younger oldest-old (aged 80–89 years) for all-cause mortality prediction. The smaller estimates among relatively older adults may be partially caused by survival bias [28]. Our study was focused on the Chinese oldest-old; therefore, this association warrants further investigation in other ethnic and age groups.

The nationally representative sample of the oldest-old population in China and the availability of blood samples in a relatively large sample size provided a unique opportunity to estimate the interrelationships among hs-CRP levels, cognitive function and all-cause mortality in the oldest-old [24]. The comprehensive information on self-reported history of chronic diseases and physical examinations at baseline made it possible to control the potential confounding bias of these covariates on mortality [28].

The current study nevertheless had several limitations. Firstly, our study had only one measure of inflammation, hs-CRP, that may be subject to the bias of regression dilution; the presence of regression dilution would be to attenuate the effect estimates and therefore could mean that our results underestimate the association of hsCRP and mortality [40]. Furthermore, the results were not changed much by excluding the participants with hs-CRP level higher than 10 mg/L; this is supportive evidence that our results are not chance findings. Secondly, we could not ignore the potential overadjustment bias as some of the chronic diseases may be mediators-on the causal pathways from exposures (hs-CRP or cognition) to outcome (mortality)-but not confounders, which would bias the risk estimates to the null. Although the results were nearly identical after these chronic diseases were excluded from the adjusted models, the complex causal pathways in relation to survival with multiple confounders and mediators warranted further investigation. Thirdly, cause-specific death data were not available in our study so we could not compare whether the HRs of the hs-CRP/cognition combined effects were significantly different between vascular and non-vascular mortality [41].

## Conclusion

In conclusion, we found that the oldest-old in China with concurrently elevated hs-CRP and cognition impairment were at the highest risk of all-cause mortality. The combined effect was stronger in male and younger oldest-old for all-cause mortality prediction. Additional research is needed to describe both the specific and combined effects of inflammation and cognition on mortality as well as the biological mechanism underlying these associations.

## Supplementary information

**Additional file 1: Table S1.** Baseline characteristics of study participants across the 4 groups (*N*=1447). **Table S2.** Baseline characteristics of study participants by follow-up status (*N*=1447). **Table S3.** Hazard ratios for the individual associations of hs-CRP level and cognitive function with all-cause mortality (*N*=1447). **Table S4.** Hazard ratios for the combined associations of hs-CRP and cognitive impairment with all-cause mortality, without adjustment for chronic diseases (*N*=1447).

## Data Availability

The datasets used and/or analyzed during the current study are available from the corresponding author on reasonable request.

## Abbreviations

BMI: Body mass index
CI: Cognitive impairment
CLHLS: Chinese Longitudinal Healthy Longevity Survey
HRs: Hazard ratios
Hs-CRP: High sensitivity C-reactive protein
MMSE: Mini-Mental Status Examination
SD: standard deviation
WC: Waist circumference
