# Supplementary material for: Combined associations of hs-CRP and cognitive function with all-cause mortality among oldest-old adults in Chinese longevity areas: a prospective cohort study
Source: Immun Ageing. 2019 Nov 17;16:30. doi: 10.1186/s12979-019-0170-y (PMC6859603; doi:10.1186/s12979-019-0170-y)
Supplement: Supplementary file 1 — Additional file 1: Table S1. Baseline characteristics of study participants across the 4 groups (N=1447). Table S2. Baseline characteristics of study participants by follow-up status (N=1447). Table S3. Hazard ratios for the individual associations of hs-CRP level and cognitive function with all-cause mortality (N=1447). Table S4. Hazard ratios for the combined associations of hs-CRP and cognitive impairment with all-cause mortality, without adjustment for chronic diseases (N=1447). [file 12979_2019_170_MOESM1_ESM.docx]

Table S1. Baseline characteristics of study participants across the 4 groups (N=1447)

| Characteristic | Overall^a^  (n=1447) | Group 1 (n=689) | Group 2 (n=220) | Group 3 (n=389) | Group 4 (n=149) | P for trend |
| --- | --- | --- | --- | --- | --- | --- |
| Self-reported chronic diseases | |  |  |  |  |  |
| Hypertension, yes^b^ | 887 (67.5) | 456(69.1) | 141(67.4) | 220(61.2) | 70(64.4) | 0.275 |
| Diabetes mellitus | 111 (9.2) | 46(7.2) | 22(11.6) | 29(8.9) | 14(27.4) | 0.016 |
| Heart Disease, yes^b^ | 94 (10.0) | 56(11.3) | 14(10.5) | 18(2.4) | 6(9.0) | 0.128 |
| Stroke/CVD, yes^b^ | 117 (10.9) | 40(6.5) | 22(13.0) | 34(24.3) | 21(27.6) | <0.001 |
| Respiratory disease, yes^b^ | 118 (9.9) | 54(9.0) | 27(15.0) | 28(6.3) | 9(9.1) | 0.996 |
| Cancer, yes^b^ | 6 (0.5) | 2(0.4) | 2(0.9) | 2(0.4) | / | / |

Note: Values are given as No.(%) unless otherwise stated. No. was based on study samples (unweighted). Means (SD) and percentages were weighted population estimates. Group 1 (hs-CRP≤3.0 mg/L and normal cognition), group 2 (hs-CRP>3.0 mg/L and normal cognition), group 3 (hs-CRP≤3.0 mg/L and CI), group 4 (hs-CRP>3.0 mg/L and CI).

^a^Of 1447 decedents, 46 with missing data on weight variable were excluded for calculating weighted population estimates. ^b^Numbers of missing data ranged from 1 to 54 (1 for hypertension, 36 for heart disease, 28 for Stroke/CVD, 28 for respiratory disease, and 54 for cancer).

Table S2. Baseline characteristics of study participants by follow-up status (N=1447).

| Characteristic^a^ | Successfully followed-up (n=1121) | Lost to follow-up (n=287) | P-value |
| --- | --- | --- | --- |
| Age, years, mean (SD) | 84.6(0.2) | 84.9(0.3) | 0.204 |
| Female | 722(56.8) | 177(65.7) | 0.086 |
| ≥1 Years of education | 237(30.5) | 89(32.3) | 0.710 |
| Currently married, yes^b^ | 234(38.6) | 63(35.5) | 0.574 |
| Regular exercise, yes^b^ | 125(13.0) | 57(27.4) | <0.001 |
| Current smoking, yes^b^ | 114(12.2) | 37(10.1) | 0.455 |
| Current alcohol drinking, yes^b^ | 137(13.6) | 32(10.7) | 0.390 |
| Body mass index, kg/m^2^, mean (SD) | 21.2(0.4) | 22.0(0.7) | 0.198 |
| Central obesity, yes^bc^ | 385(41.2) | 80(34.6) | 0.244 |
| Adequate medical service, yes^b^ | 1043(93.5) | 268(96.7) | 0.197 |
| Hypertension, yes^b^ | 707 (67.6) | 180(67.3) | 0.945 |
| Diabetes mellitus | 88(7.9) | 23(13.9) | 0.072 |
| Heart Disease, yes^b^ | 80 (10.6) | 14(7.7) | 0.409 |
| Stroke/CVD, yes^b^ | 97 (11.7) | 20(8.0) | 0.267 |
| Respiratory disease, yes^b^ | 93 (9.9) | 25(9.7) | 0.950 |
| Cancer, yes^b^ | 6 (0.6) | / | / |

Note: Values are given as No. (%) unless otherwise stated. No. was based on study samples (unweighted). Means (SD) and percentages were weighted population estimates.

^a^Of 1447 decedents, 46 with missing data on weight variable were excluded for calculating weighted population estimates. ^b^Numbers of missing data ranged from 1 to 54 (8 for married status, 41 for regular exercise, 8 for current smoking, 3 for alcohol drinking, 18 for central obesity, 6 for adequate medication, 1 for hypertension, 36 for heart disease, 28 for Stroke/CVD, 28 for respiratory disease, and 54 for cancer).

Table S3. Hazard ratios for the individual associations of hs-CRP level and cognitive function with all-cause mortality (N=1447).

| hs-CRP(mg/L) | Model 1 | Model 1+cognition | Model 2 | Model 2+cognition^*^ |
| --- | --- | --- | --- | --- |
| Each 1 mg/L increment | 1.02 (0.99, 1.02) | 1.01 (1.00, 1.02) | 1.00 (0.99, 1.02) | 1.01 (1.00, 1.02) |
| Cognitive function | Model 1 | Model 1+hs-CRP | Model 2 | Model 2+hs-CRP |
| Each 1-unit score increment | 0.94 (0.92, 0.95) | 0.94 (0.92, 0.95) | 0.95 (0.93, 0.96) | 0.95 (0.93, 0.96) |

Model 1 adjusted for age and sex; model 2 further adjusted for education, drinking, smoking, marital status, regular exercise, medication, BMI, central obesity, self-reported history of hypertension, diabetes mellitus, heart disease, stroke and cerebrovascular disease, respiratory disease and cancer.

*Education was not included.

Table S4. Hazard ratios for the combined associations of hs-CRP and cognitive impairment with all-cause mortality, without adjustment for chronic diseases (N=1447).

| Groups/HR | No. of deaths | Model 1 | Model 2^a^ |
| --- | --- | --- | --- |
| 1: hs-CRP≤3.0 mg/L and normal cognition | 312 | 1 | 1 |
| 2: hs-CRP>3.0 mg/L and normal cognition | 117 | 1.80 (1.24, 2.61) | 1.74 (1.17, 2.59) |
| 3: hs-CRP≤3.0 mg/L and CI | 286 | 2.79 (1.82, 4.28) | 2.35(1.54, 3.57) |
| 4: hs-CRP>3.0 mg/L and CI | 111 | 4.61 (3.16, 6.72) | 3.64 (2.39, 5.54) |
| P for trend | - | <0.001 | <0.001 |

Model 1 adjusted for age and sex; model 2 further adjusted for drinking, smoking, marital status, regular exercise, medication, BMI, central obesity. CI=cognitive impairment

^a^ P for interaction<0.01.
